# Secondary Effects of Antibiotics on Microbial Biofilms

**DOI:** 10.3389/fmicb.2020.02109

**Published:** 2020-09-02

**Authors:** Anahit Penesyan, Ian T. Paulsen, Michael R. Gillings, Staffan Kjelleberg, Michael J. Manefield

**Affiliations:** ^1^School of Chemical Engineering, University of New South Wales, Sydney, NSW, Australia; ^2^Department of Molecular Sciences, Faculty of Science and Engineering, Macquarie University, Sydney, NSW, Australia; ^3^ARC Centre of Excellence in Synthetic Biology, Macquarie University, Sydney, NSW, Australia; ^4^Department of Biological Sciences, Faculty of Science and Engineering, Macquarie University, Sydney, NSW, Australia; ^5^Singapore Centre for Environmental Life Sciences Engineering, Singapore, Singapore; ^6^School of Biological Sciences, Nanyang Technological University, Singapore, Singapore; ^7^School of Biological, Earth and Environmental Sciences, University of New South Wales, Sydney, NSW, Australia; ^8^School of Civil and Environmental Engineering, University of New South Wales, Sydney, NSW, Australia

**Keywords:** antimicrobials, subinhibitory, antibiotic resistance, infection, environmental pollution

## Abstract

Biofilms are assemblages of microorganisms attached to each other, or to a surface, and encased in a protective, self-produced matrix. Such associations are now recognized as the predominant microbial growth mode. The physiology of cells in biofilms differs from that of the planktonic cells on which most research has been conducted. Consequently, there are significant gaps in our knowledge of the biofilm lifestyle. Filling this gap is particularly important, given that biofilm cells may respond differently to antibiotics than do planktonic cells of the same species. Understanding the effects of antibiotics on biofilms is of paramount importance for clinical practice due to the increased levels of antibiotic resistance and resistance dissemination in biofilms. From a wider environmental perspective antibiotic exposure can alter the ecology of biofilms in nature, and hence disrupt ecosystems. Biofilm cells display increased resilience toward antibiotics. This resilience is often explained by mechanisms and traits such as decreased antibiotic penetration, metabolically inactive persister cells, and intrinsic resistance by members of the biofilm community. Together, these factors suggest that cells in biofilms are often exposed to subinhibitory concentrations of antimicrobial agents. Here we discuss how cells in biofilms are affected by the presence of antibiotics at subinhibitory concentrations, and the possible ramifications of such secondary effects for healthcare and the environment.

## Introduction

Biofilms are usually defined as microbial assemblages that can be either surface-associated, or present as an unattached but aggregated cell biomass. Biofilm cells are encased within a self-produced matrix comprised of extracellular polymeric substances (EPS) including polysaccharides, extracellular DNA (eDNA), proteins and lipids. The EPS matrix provides protection for the cells inside biofilms by acting as a shield, and is central to biofilm structure and integrity (Flemming and Wingender, 2010).

Biofilms are recognized as the predominant form of bacterial and archeal life. While the evaluation of biofilm vs. planktonic lifestyle is inconclusive for oceanic environments due to common sampling techniques that do not distinguish between the two lifestyles, more accurate estimates suggest that on the surface of the Earth biofilms dominate all habitats and account for ∼80% of bacterial and archeal cells (Flemming and Wuertz, 2019). Biofilms are common both in nature and in association with host organisms. They protect bacteria from external threats including protozoan grazing (Matz et al., 2005) and antibiotic exposure (Penesyan et al., 2015).

Biofilm communities are important for ecosystems. They drive biogeochemical processes and play fundamental roles in nutrient cycling and bioremediation (Yadav, 2017; Flemming and Wuertz, 2019). Biofilms are also an essential part of human, animal and plant microbiomes (de Vos, 2015). However, both in the environment and in association with a host, biofilm formation by bacteria can pose major challenges and risks.

Biofilms are the leading cause of biomass build-up on submerged surfaces (pipes, catchments, filters, ship hulls), becoming reservoirs for infection and also resulting in equipment failure and decreased productivity. Since biofilms are extremely resilient to external stressors, such as antimicrobials, they are difficult to eradicate. Thus, biofilms are estimated to cost billions of dollars to the United States industry alone, in the maintenance and replacement of essential infrastructure such as water transmission and distribution networks, as well as in the maintenance and cleaning of ship hulls (U.S. EPA, 2002; Schultz et al., 2011).

More than 60% of all infections in hospitals and healthcare settings world-wide are due to bacterial pathogens that form biofilms (Moscoso et al., 2009). Due to the additional layer of protection provided by the biofilm lifestyle, biofilm bacteria can display an increase in antibiotic resistance by orders of magnitude compared to their planktonic state, and are therefore a major obstacle to infectious disease treatment (Penesyan et al., 2015). As a result, nearly 80% of biofilm-related infections do not respond to antibiotic treatment (Moscoso et al., 2009) leading to increased healthcare costs and patient mortality/morbidity. Estimates suggest that biofilm infections cost nearly $100 billion annually to the United States healthcare system and cause more than 500,000 deaths in the United States alone (Wolcott et al., 2010).

Many antibiotics derive from natural products found in environmental ecosystems. Bacteria have hence had an opportunity to encounter these compounds during billions of years of evolution. Consequently, microorganisms have adapted by arming themselves with strategies to overcome toxic effects of antibiotics. Moreover, microbes can use antibiotics as cues or signals, often developing physiological responses that provide them with an ecological advantage that increases their survival (Linares et al., 2006).

The ability to control biofilm formation is of great importance in medicine and industry. As a result, the effects of antibiotics on biofilms have largely focused on the recalcitrance of biofilms toward antimicrobials, and the development of tools to reduce that resilience, thus making treatments more effective. In this review we focus on rarely considered but important effects that antimicrobials have on biofilm cells at subinhibitory concentrations. This approach can help to better understand the roles of antimicrobials in biofilm biology, and to recognize possible unintended consequences of our current strategies to control infections and biofilm growth.

## Biofilms in Infections

Antibiotics can have a variety of effects on microbial biofilms. These effects can be complex and depend on many factors, such as the concentration of antibiotics to which the organisms are exposed, specific growth conditions, and the characteristics of the organism itself. The effect of antibiotics on biofilms in infections can be roughly evaluated as two seemingly opposing effects: disrupting existing biofilms or enhancing biofilm formation.

### Disrupting Biofilms: A Double-Edged Sword

The biofilm-disruptive effects of some antibiotics can seem promising for antibiotic therapy. Destabilization of the biofilm structure can be regarded as a way to eliminate the additional protection provided by biofilms, thus making the biofilms more penetrable for antimicrobials, and biofilm cells more susceptible to antibiotics. However, this strategy is fraught with peril as biofilm structure destabilization and loosening of the biofilm matrix can lead to cellular detachment and further dissemination of biofilms, potentially leading to increasingly severe and long-term consequences.

In a recent study Díaz-Pascual et al. (2019) examined the effects of antibiotics commonly used to treat cholera on biofilms of *Vibrio cholerae*. Transient exposure to translation-inhibiting antibiotics such as tetracycline caused alterations in cell shape and physiology that resulted in large-scale changes in biofilm architecture and the dismantling of cell-matrix associations. This effect may be considered favorable for *V. cholerae* biofilm eradication, as loosening of biofilm structures may allow antimicrobials better access to the biofilm interior. However, disrupting the biofilm structure may detach cells that can then serve as inocula for new points of infection, and hence cause spreading of the infectious agent.

Clinically relevant concentrations of colistin, a polymyxin antibiotic that primarily targets Gram-negative bacteria via disrupting bacterial membranes, have been found to also destabilize the biofilm matrix of clinical isolates of the Gram-positive pathogen *Staphylococcus aureus* as well as Gram-negative *Escherichia coli* (Klinger-Strobel et al., 2017). The electrostatic interaction of the amphiphilic molecule colistin with matrix exopolysaccharides was suggested as a possible mechanism. Similar effects were also observed when biofilms of the opportunistic pathogen *Pseudomonas aeruginosa* were exposed to subinhibitory concentrations of the cation-chelator antibiotic nitroxoline (Sobke et al., 2012).

Yoshii et al. (2017) have shown that norgestimate, a compound primarily used for oral contraception and hormone replacement therapy (Henzl, 2001) but also investigated as a potential antibiofilm/antibiotic agent, was able to inhibit biofilm formation by staphylococcal strains. It did so via inhibiting production of a polysaccharide adhesin and proteins in the extracellular matrix, thus affecting biofilm structure and integrity (Yoshii et al., 2017).

Likewise, *Enterococcus faecalis*, an opportunistic pathogen that is the most prevalent enterococcal species identified in healthcare-associated infections, displayed changes in biofilm architecture and restructuring of biofilm monolayers into complex three-dimensional biofilms upon the exposure to subinhibitory concentrations of clinically relevant antibiotics. This effect was seen across antibiotics with diverse modes of action, including daptomycin (disrupts bacterial cell membranes) and gentamicin (protein synthesis inhibitor) (Dale et al., 2017). The effect was related to the cellular stress response and was accompanied by increased biofilm detachment. While this response could make these bacteria more susceptible to antibiotics or to immune responses, biofilm detachment could also be advantageous for *E. faecalis* in terms of dissemination and long-term reproduction during infection.

### Enhancing Biofilm Formation and Resilience

There is mounting evidence (reviewed in Kaplan, 2011) to suggest that subinhibitory concentrations of many antibiotics are able to enhance biofilm formation by pathogens.

Exposure of *Campylobacter jejuni*, a bacterium often associated with human gastroenteritis, to various antibiotics led to enhanced biofilm formation in antibiotic susceptible strains, suggesting that the response reflects a survival strategy by these strains (Teh et al., 2019). A similar biofilm enhancing effect was observed for *Leptospira* spp. after treatment with subinhibitory concentrations of the antibiotics doxycycline and tetracycline, the drugs of choice for leptospirosis (Vinod Kumar et al., 2018).

The macrolide antibiotic azithromycin (protein synthesis inhibitor) is reported to interfere with quorum-sensing-dependent virulence factor production, biofilm formation, and oxidative stress resistance in *P. aeruginosa*. This organism is a major bacterial pathogen associated with cystic fibrosis chronic infections, and azithromycin treatment led to improved patient outcomes (Equi et al., 2002; Saiman et al., 2003; Nalca et al., 2006). However, while the antibiotic was effective during its first year of administration, it showed a poor efficacy thereafter (Tramper-Stranders et al., 2007; Principi et al., 2015; Willekens et al., 2015). Using *in vitro P. aeruginosa* biofilms, it was demonstrated that azithromycin delayed biofilm formation, but these effects were short-lived and appeared to be specific to the initial stages of biofilm development. After 48 h, an emerging resistant phenotype was able to overcome the inhibitory effect of azithromycin and result in a very robust biofilm (Gillis and Iglewski, 2004). Thus, the limited long-term effect of azithromycin on *in vitro* microbial biofilms may explain its failure in the prolonged treatment of cystic fibrosis infections in clinical practice.

Subinhibitory concentrations of aminoglycoside antibiotics (protein synthesis inhibitors) were found to induce biofilm formation in the opportunistic human pathogens *P. aeruginosa* and *E. coli* (Hoffman et al., 2005). This effect was described as a specific defensive reaction to the presence of antibiotics and was linked to alterations in the level of c-di-GMP, an important bacterial second messenger that regulates cell surface adhesiveness, biofilm formation and virulence (D’Argenio and Miller, 2004; Ryan, 2013). In addition, increased biofilm formation in *E. coli* upon antibiotic exposure has been shown for the exposure to the bacterial cell-wall targeting antibiotics colistin and carbenicillin and for the translation-inhibiting drug tetracycline (Boehm et al., 2009). A biofilm-enhancing effect of tetracycline was also recently demonstrated for the nosocomial pathogen *Acinetobacter baumannii*, as a response to low-level antibiotic exposure, and was accompanied by rapid genome evolution and the generation of antibiotic-resistant mutants (Penesyan et al., 2019).

The close proximity of microbes within biofilm aggregates and the abundance of eDNA may facilitate horizontal gene transfer and the spread of resistance determinants (Penesyan et al., 2015). Exposure to subinhibitory antibiotic concentrations can promote the growth of resistant and/or more fit variants by both selecting for preexisting mutants and by promoting new mutations (Andersson and Hughes, 2014; Ahmed et al., 2018; Santos-Lopez et al., 2019). A recent study demonstrated that such effects can be extraordinarily rapid in biofilms. Within 3 days of exposure, subinhibitory antibiotic concentrations can help generate and select an array of mutations that confer resistant phenotypes (Penesyan et al., 2019).

The biofilm lifestyle and the presence of antibiotics are known to induce the bacterial stress response, including the general stress response, oxidative stress response accompanied by the induction of central carbon metabolites and elevated TCA cycle intermediates, stringent response and the SOS response. The latter increases mutation rates and facilitates horizontal transfer of DNA, including antimicrobial resistance determinants (Kohanski et al., 2010; Gutierrez et al., 2013; Belenky et al., 2015; Strugeon et al., 2016; von Wintersdorff et al., 2016; Gillings, 2017; Liu et al., 2019). Thus, biofilms may constitute specific foci of genetic adaptation and evolution, leading to the selection of subpopulations with a greater ability to withstand current and future antibiotic exposure.

Antibiotic exposure can also lead to transient adaptive physiological changes. *Streptococcus intermedius*, a commensal bacterium associated with periodontitis, fatal purulent infections and brain and liver abscesses, showed increased biofilm formation upon exposure to subinhibitory concentrations of three antibiotics with distinct modes of action and different bacterial targets. These included ampicillin (bacterial cell wall synthesis inhibitor), ciprofloxacin (transcription inhibitor), and tetracycline (translation inhibitor). The increased biofilm formation was linked to the *S. intermedius* AI-2/LuxS quorum sensing system being activated in the presence of antibiotics (Ahmed et al., 2009). In contrast, ciprofloxacin, as well as azithromycin (protein synthesis inhibitor) and ceftazidime (bacterial cell wall inhibitor), were found to decrease the expression of quorum-sensing related genes in *P. aeruginosa* (Skindersoe et al., 2008). These suggest that such effects can be both strain- and antibiotic-specific, and, therefore, hard to predict.

Changes in the morphology of biofilm cells (i.e., rounding, blebbing, and alteration of cell size) were observed in *Klebsiella pneumoniae* biofilms in the presence of sublethal concentrations of β-lactam carbapenems (cell wall inhibitors) imipenem, meropenem and doripenem (Van Laar et al., 2015). In general, despite morphological differences observed, there were no significant differences in viability in most treated biofilms, with the exception of a slight decrease in viability observed in doripenem-treated biofilm cells. Cells in biofilms treated with all three antibiotics returned to their normal pre-treatment morphology soon after the removal of the antibiotic and showed no significant change in viability. A number of genes were implicated in these morphological transitions, including genes involved in peptidoglycan biosynthesis and catabolism. Genes involved in the general stress response, virulence and antibiotic resistance were differentially expressed in the presence of imipenem, causing profound changes in cellular physiology. Upregulation of genes involved in the formation of antibiotic tolerant persister cells was also observed in imipenem-treated biofilms, possibly contributing to the ability of these biofilms to remain viable even with a carbapenem treatment of 1,000× the MIC (Minimum Inhibitory Concentration, as tested in planktonic cultures) (Van Laar et al., 2015).

Exposure to the β-lactam antibiotic imipenem induced genes coding for alginate biosynthesis in *P. aeruginosa* and led to increased biofilm volume (Bagge et al., 2004). Alginate is an important polysaccharide component of *P. aeruginosa* biofilms that serves to protect bacteria from adverse conditions while also enhancing its adhesion to surfaces (Boyd and Chakrabarty, 1995). Alginate production by *P. aeruginosa* underlies its conversion to mucoid phenotype and is linked to the development of impaired lung function in cystic fibrosis patients (Li et al., 2005). The increased expression of alginate in response to imipenem may be an unintended adverse consequence of imipenem treatment of *P. aeruginosa* infections in cystic fibrosis (Bagge et al., 2004).

Beta-lactam antibiotics methicillin, ampicillin, amoxicillin, and cloxacillin were shown to induce biofilm formation in *Staphylococcus aureus* strains, including methicillin-resistant *S. aureus* (MRSA). This effect was linked to autolysin-dependent release of eDNA, an important constituent of biofilms (Kaplan et al., 2012). Likewise, Yu et al. (2018) demonstrated that subinhibitory concentrations of bacterial cell-wall targeting antibiotics led to enhanced biofilm formation and increased density of biofilm cells in the prominent nosocomial pathogen *E. faecalis*. This effect was associated with increased cell lysis accompanied by a surge in eDNA levels, suggesting that such effects may take place in a variety of Gram-positive pathogens.

*Bacillus subtilis* was shown to form biofilms in response to a variety of structurally dissimilar bioactive natural products of microbial origin, including those produced by *B. subtilis* itself. It was suggested that these molecules cause potassium leakage across the cytoplasmic membrane. This results in the activation of a protein kinase that sets in motion a chain of regulatory events, inducing the expression of genes involved in the synthesis of EPS matrix constituents – a central component of the biofilm architecture (López et al., 2009). A similar biofilm-enhancing effect in *B. subtilis* was demonstrated for a variety of thiopeptide antibiotics known to interfere with ribosomal functioning. This suggests that, besides their anthropogenic application for inhibiting bacterial growth, antibiotics may have complex roles as signaling molecules that specifically modulate bacterial phenotypes (Bleich et al., 2015; Townsley and Shank, 2017).

The notion that antibiotics have intrinsic environmental roles and can act as signals in diverse biological processes is not novel (Yim et al., 2007). Linares et al. (2006) demonstrated a signaling role for antibiotics by showing the biofilm enhancing effects of several antibiotics on the important opportunistic pathogen *P. aeruginosa*, accompanied by changes in physiology and ecological behavior. Thus, in such scenarios, at subinhibitory levels the antibiotics become beneficial to the pathogen, promoting its survival, hence having the opposite effect to their intended use as growth inhibitors.

The impacts of subinhibitory concentrations of antibiotics on a range of phenotypic outcomes, including enhanced survival, complicate biofilm eradiction efforts. Rather than achieving biofilm removal, antibiotics may also strengthen and enhance the survival of microbes by increasing the protection provided by biofilms.

## Biofilms in Nature

There is a continuous discharge of effluents from human activities, including industrial and hospital waste, into water reservoirs such as rivers and lakes. Along with wastewater, antibiotics used in clinical practice and agriculture often end up in environmental reservoirs and in coastal marine environments (Nödler et al., 2014; Nakayama et al., 2017). Here, they add to the burden of recalcitrant anthropogenic pollutants known as Trace Organic Compounds (TOrCs) (Basiuk et al., 2017). Microorganisms inhabiting polluted water bodies are consequently exposed to varying low concentrations of chemical pollutants, including antibiotics (Yadav, 2017; Chow et al., 2021).

Biofilms associated with infections in the human body tend to involve a limited number of species (Burmølle et al., 2010). In contrast, environmental biofilms are often diverse communities that include multiple taxonomic groups with a multitude of functions. Compared to the wealth of research data on antibiotics and their effects on biofilms in clinical settings, data on environmental biofilms are very limited.

As discussed above, antibiotics have diverse effects on various biofilm communities, including enhanced biofilm formation, increased resistance and the spread of antimicrobial resistant phenotypes and genotypes. The increasing presence of antibiotic compounds in the environment is therefore of great concern. Environmental biofilms downstream of a sewer overflow site exhibit increased antibiotic resistance (Kaeseberg et al., 2018). Moreover, antibiotic resistance genes and resistant organisms are often found in natural waterways and can also persist for prolonged periods of time within sediments (Calero-Cáceres et al., 2017; Zhu et al., 2017; Gillings, 2018; Guo et al., 2018; Lépesová et al., 2018). Thus, these environments can serve as reservoirs for antibiotic resistant organisms and further facilitate dissemination of resistance genes.

Triclosan is a bactericidal compound that has been widely used in cosmetics and household cleaners. It is able to enter fluvial ecosystems after surviving the degradation steps in wastewater treatment plants (Ricart et al., 2010). Short-term effects of triclosan on fluvial biofilms caused bacterial mortality and the inhibition of photosynthetic activity in autotrophs (Ricart et al., 2010). Another study, however, showed that despite initial widespread bacterial mortality, laboratory-grown multispecies stream biofilms recovered their normal structure and function within a few days or weeks after exposure, highlighting the capacity of biofilms to tolerate and persist in the face of periodic inputs of toxic compounds (Proia et al., 2011).

In another study, Proia et al. (2013) investigated the effects of antibiotics on biofilm communities in the Llobregat River (Northeast Spain) where the antibiotics present as pollutants at the highest concentrations were sulfonamides, followed by quinolones and macrolides. An enhanced bacterial mortality, an increased abundance of *Actinobacteria* and a general decrease of enzymatic activity in biofilms exposed to the polluted water were documented. This indicates that the release of antibiotics into running waters can cause significant structural and functional changes in microbial biofilm communities.

As environmental biofilm communities serve an essential role in ecosystem health and functioning, antibiotic driven disturbances of these communities may have unexpected consequences. In biofilm communities involved in anaerobic ammonium oxidation (annamox), a globally important microbial process that is used in wastewater treatment, the presence of trace amounts of norfloxacin significantly suppresses annamox activity (Zhang X. et al., 2019).

Biofilms themselves can also affect the fate of antibiotics and their impacts in the environment. For example, a recent study has shown that sorption of the fluoroquinolone antibiotic ofloxacin to sediments was inhibited when sediments were coated by biofilms, increasing the concentration of the antibiotics in the aqueous phase and further enhancing the ecological risks associated with the presence of the antibiotic in water reservoirs (Zhang L. et al., 2019).

## Conclusions and Future Perspectives

**FIGURE 1:**
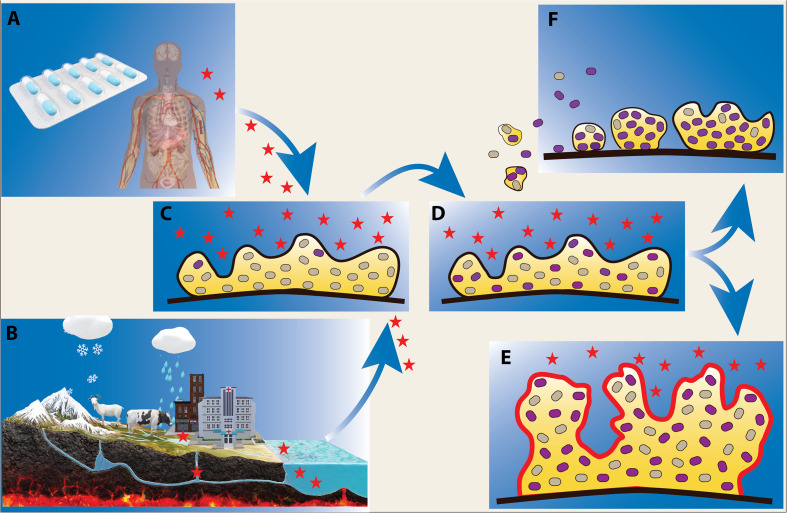
Effect of low levels of antibiotics on microbial biofilms. Biofilm bacteria are often exposed to low levels of antibiotics during infection treatment and antibiotic therapy **(A)**, and in natural water reservoirs where there is a flux of antibiotics from sources such as agriculture, aquaculture, hospital and domestic waste **(B)**. Exposure of biofilms to antibiotics (indicated as red stars) **(C)** can lead to the emergence of antibiotic resistant phenotypes/genotypes **(D)** (shown as purple cells) and facilitate formation of more robust biofilms **(E)**. Upon antibiotic exposure, resistant biofilm bacteria can further disseminate and serve as inocula for potentially more resistant biofilm communities **(F)**.

Studying the potential consequences of antibiotic discharge into the environment is of paramount importance for evaluating short-term and long-term effects of antibiotic use. The sources of such antibiotic pollution are diverse, and include hospitals, industry, agriculture, aquaculture and domestic use (Figure 1).

The primary application of antibiotics has been at growth-inhibitory concentrations, and research aimed at disclosing the mode of action and outcomes provided by antibiotics has generally been conducted at such concentrations. However, data show that depending on concentration, antibiotic exposure can trigger different physiological responses. Subinhibitory concentrations, in particular, can enhance the growth and resilience of biofilms and promote the dissemination of cells from existing biofilms. Therefore, measures need to be taken to limit unintentional exposure of microbial biofilms to low levels of antibiotics, both in healthcare and environmental settings.

The excessive and often unnecessary use of antibiotics has become a worldwide problem for healthcare as it drives antibiotic resistance in pathogens and hinders our ability to treat infections. Combatting infectious diseases is especially problematic in developing countries with limited or inadequate access to medications. This problem is exacerbated by the potential use of counterfeit and substandard antibiotic preparations. The most common types of substandard/counterfeit antimicrobial drugs have a reduced amount of the active drug (Kelesidis and Falagas, 2015). The use of such preparations leads to the exposure of pathogens in biofilms to sublethal doses of antimicrobials with potentially far reaching consequences. Better controls of antibiotic usage and substandard/counterfeit antibiotic preparation may help to ameliorate some of the problems discussed above.

The use of proper antibiotic waste management procedures, as well as strategies to reduce the presence of antibiotics in environmental reservoirs using managing aquifer recharge (MAR) systems (Drewes, 2009) and various other biotic (e.g., via microbial degradation) and abiotic (e.g., via sorption, hydrolysis, photolysis etc.) remediation procedures (Kumar et al., 2019) are also necessary to limit the exposure of microbial biofilms to antibiotics and minimize their subsequent secondary effects. To this end much additional work is required to understand the environmental fate as well as biodegradation processes of antibiotics, in order to reduce contaminant loads in the environment.

## Author Contributions

All authors have made a direct intellectual contribution to the work and approved it for publication.

## Conflict of Interest

The authors declare that the research was conducted in the absence of any commercial or financial relationships that could be construed as a potential conflict of interest.
